# Genome-Wide Analysis of the Rice PcG Gene Family and Its Involvement in Salt Response and Development

**DOI:** 10.3390/plants14172805

**Published:** 2025-09-08

**Authors:** Ziang Shi, Jun Cao, Chuheng Li, Jun Liu, Xinlei Yang, Xiliu Cheng

**Affiliations:** 1 State Key Laboratory of North China for Crop Improvement and Regulation, North China Key Laboratory for Crop Germplasm Resources of Education Ministry, Key Laboratory of Crop Germplasm Resources of Hebei Province, Hebei Agricultural University, Baoding 071001, China; ziang1031@163.com (Z.S.); lichuheng5377@foxmail.com (C.L.); 2 Institute of Life Science and Green Development/Hebei Basic Science Center for Biotic Interaction, College of Life Science, Hebei University, Baoding 071002, China; caojun@stumail.hbu.edu.cn; 3 Institute of Crop Sciences, Chinese Academy of Agricultural Sciences, Beijing 100081, China; liujun@caas.cn

**Keywords:** *Oryza sativa*, Polycomb group proteins, *OsPcG* genes, PRC2 complex, salt stress, epigenetic regulation, gene expression, chromatin modification

## Abstract

Polycomb group (PcG) proteins are pivotal in maintaining gene silencing through epigenetic mechanisms, particularly by catalyzing Histone H3 lysine 27 trimethylation (H3K27me3) via the Polycomb Repressive Complex 2 (PRC2) complex. These modifications are crucial for regulating developmental pathways and environmental stress responses in plants. Despite their importance, the PcG gene family has not been systematically explored in rice (*Oryza sativa*). In this study, 15 *OsPcG* genes were identified in the Nipponbare genome, spanning 12 chromosomes and classified into distinct phylogenetic groups. Structural and conserved motif analyses revealed high sequence conservation, while collinearity and Ka/Ks analyses indicated gene family expansion through segmental duplication under purifying selection. Promoter element prediction suggested that many *OsPcG* genes are responsive to plant hormones and abiotic stress cues. Transcriptome analysis under salt treatment highlighted *OsPcG5* as a key salt-responsive gene, with qRT-PCR confirming its dynamic expression. Subcellular localization showed *OsPcG5* residing in both the nucleus and plasma membrane, suggesting multifunctional roles. Additionally, overexpression of *OsFIE2*—a PRC2 component—resulted in elevated H3K27me3 levels and abnormal plant height, linking it to chromatin modification and development. These findings contribute to our understanding of *PcG* gene functions in rice and offer potential genetic resources for enhancing salt tolerance through epigenetic approaches.

## 1. Introduction

Polycomb group (PcG) proteins maintain long-term transcriptional repression by modifying chromatin architecture, thereby preserving cellular identity through stable gene silencing mechanisms [1,2]. The core mechanisms of epigenetic inheritance include DNA methylation, histone modification, and non-coding RNA regulation, each of which affects gene expression in different ways [3].

In particular, DNA methylation is the addition of methyl groups (usually occurring on cytosine) to a DNA molecule, thereby altering the expression status of a gene. Usually, the modification occurs in the CpG island region, and high levels of DNA methylation are often associated with gene silencing, while hypomethylation may lead to aberrant gene expression [4]. In addition to DNA methylation, histone modification is also an important way to influence gene transcription. Histones are the basic building blocks of chromatin, and they can undergo a variety of chemical modifications, such as acetylation, methylation, and phosphorylation, which regulate gene activity by altering chromatin structure. In plants, the regulation and transition of several developmental processes, such as cell differentiation, organ germination, and flower morphogenesis, are controlled by chromatin modifying factors [5]. In general, histone acetylation is usually associated with gene transcriptional activation, while the role of histone methylation depends on the specific type and site of modification. For example, H3K4me3 modification is usually associated with gene activation, whereas H3K27me3 modification often leads to transcriptional repression [6]. In recent years, the role of non-coding RNAs in epigenetic regulation has received extensive attention. While microRNAs (miRNAs) regulate gene expression by targeting mRNAs and inhibiting their translation, long-chain non-coding RNAs (lncRNAs) can affect the gene transcription process by interacting with chromatin modifying factors. Studies have shown that lncRNAs are able to regulate chromatin status and thus play an important role in cell development, differentiation, and disease genesis [7]. In addition, epigenetics has been found to play an important role in the regulation of stress signaling transduction [8,9,10].

The epigenetic regulation of gene repression is often controlled by PcG proteins, which were first identified in Drosophila melanogaster [11]. PcG proteins maintain transcriptional silencing of *Hox*, a key developmental gene, through histone modifications to ensure cellular identity [12,13]. PcG proteins maintain silencing of gene expression during development through the formation of multiprotein complexes [14], which include Polycomb Repressive Complex 1 (PRC1) and Polycomb Repressive Complex 2 (PRC2) [15]. In animals, PRC2 marks silent gene loci by catalyzing histone H3K27me3 modification through its core component EZH2 (E(z) in *Drosophila*), which promotes chromatin tightening and provides the basis for PRC1 recruitment [16,17]. Subsequently, PRC1 recognizes the H3K27me3 modification and further recruits the PcG complex, which inhibits RNA polymerase II activity, thereby maintaining transcriptional silencing [18]. According to the canonical framework, PRC2 catalyzes H3K27me3 to create chromatin landscapes favorable for PRC1 binding. Nevertheless, this hierarchical recruitment model does not universally apply [19]. It has been demonstrated that RYBP-containing PRC1 complexes can associate with chromatin independently of PRC2 activity and the H3K27me3 mark [20]. It has been reported that even in the presence of PRC2 and H3K27me3, PRC1 is not always recruited to all target loci, suggesting alternative or context-dependent regulatory mechanisms.

In *Drosophila*, PRC1 contains four main subunits: Polycomb (Pc), Polyhomeotic (Ph), Posterior sex combs (Psc), and Sex Combs Extra90 (Sce) [21,22]. Polycomb (Pc) localizes PRC1 to target motifs by binding H3K27me3, Posterior sex combs (Psc) acts as an E3 ubiquitin ligase catalyzing H2A ubiquitylation modification, and Polyhomeotic (Ph) forms a stable complex with Pc and Psc. Polycomb (Pc) localizes PRC1 to the target locus by binding H3K27me3, Posterior sex combs (Psc) acts as an E3 ubiquitin ligase that catalyzes H2A ubiquitination modification, Polyhomeotic (Ph) forms a stabilizing complex with Pc and Psc and promotes chromatin aggregation, and Sex Combs Extra90 (Sce) acts as the catalytic subunit that synergizes with Psc in the H2A ubiquitination process, thus achieving transcription through histone modification and thereby realizing transcriptional repression through histone modification [18,23,24,25]. In plants, the PRC1 complex remains to be investigated. A single homolog of Pc, named Like Heterochromatin Protein1 (LHP1), was found in *Arabidopsis* (*Arabidopsis thaliana*), which acts similarly to Polycomb (Pc) [26,27]. Secondly, two homologous proteins of Sce have been identified in *Arabidopsis* as Really Interesting New Gene 1A and B (RING1A and RING1B). Both proteins contain the RING structural domain and the ubiquitin-like RAWUL structural domain, structural features that are also typical of RING1 proteins in the animal PRC1 complex, with highly conserved functions [28]. It inhibits RNA polymerase II (RNA Pol II) activity through H2AK119Ub modification to maintain transcriptional repression of target genes [29]. In addition, the genes AtBMI1A, AtBMI1B, and AtBMI1C (BMI1 stands for B lymphoma Mo-MLV insertion region 1), which are functionally similar to Psc, have been unearthed and are involved in H2A ubiquitination, similar to the H2AK119Ub modification in animals [30]. To date, no Ph-related homologs have been identified in plants [31,32].

Homologs to various components of the *Drosophila* Polycomb Repressive Complex 2 (PRC2) complex have been identified in plants. PRC2 is mainly composed of core proteins such as Enhancer of zeste E(z), Suppressor of zeste 12 (Su(z)12), Extra sex combs (Esc), and p55 (Nurf-55, Caf1-55), which are core proteins [33,34]. In plants, Fertilization-Independent Endosperm (FIE) is the only homologue of ESC in plants and plays a role in seed development, flowering time, and other growth regulation [35]. CURLY LEAF (CLF), SWINGER (SWN), and MEDEA (MEA) are all E(z) homologs [36]. They encode histone methyltransferases that catalyze the H3K27me3 modification, which is functionally similar to Drosophila E(z). FERTILIZATION-INDEPENDENT SEED 2 (FIS2), VERNALIZATION 2 (VRN2), and EMBRYONIC FLOWER 2 (EMF2) are homologs of Su(z)12 genes with similar epigenetic regulation [37,38]. In addition, MULTICOPY SUPPRESSOR OF IRA1 (MSI1) is a functional homolog of p55 that regulates chromatin state and is involved in the assembly of the PRC2 complex [39].

To date, whole-genome sequencing of rice has been completed, which provides critical support for the in-depth study of the evolutionary history and function of the rice gene family using bioinformatics. At present, studies on the genome-wide identification, evolution, and functional analysis of the rice PcG gene family are still relatively limited. Although studies have been reported to identify multiple *PcG* genes from the *Arabidopsis* genome, these genes have not been analyzed to a comprehensive and systematic extent. Therefore, in order to deeply investigate the gene structure, evolutionary relationships, and expression patterns of *PcG* genes and to assess their potential function in important biological processes, this study employed bioinformatics analysis combined with transcriptome sequencing to conduct a systematic study of the rice PcG gene family. It provides valuable basic data for further exploring the gene structure, function prediction, and expression pattern of *PcG* genes in response to abiotic stresses in rice. We identified a development and stress response-related gene *OsFIE2*, which executes the H3K27me3 modification in cells, through promoter *cis*-elements analysis. Via transcriptome and qRT-PCR analysis of salt-treated rice seedlings, *OsFIE2* was found to be induced by salt stress. Furthermore, overexpression lines of *OsFIE2* in NIP exhibit altered plant height in the seedling and adult stages. Some *OsFIE2* overexpression lines showed increased H3K27me3 levels in WB assays.

## 2. Results

### 2.1. OsPcG Identification and Chromosome Localization

**Figure 1:**
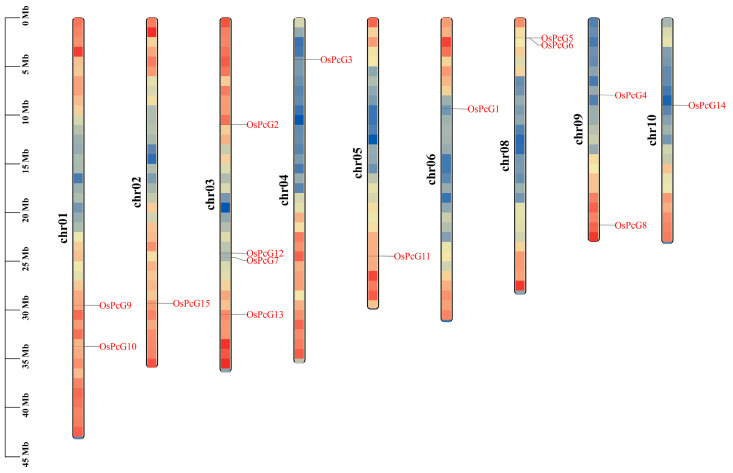
*OsPcG* chromosome localization. The color gradient represents gene density, with red indicating high density and blue indicating low density.

The rice native protein database was searched using the HMM profile containing the PcG structural domain to identify *PcG* genes in cultivated rice. A total of 15 *OsPcG* genes, named *OsPcG1* to *OsPcG15*, were identified (Table S1 and Figure 1). All the members contained characteristic structural domains that could be further analyzed. The *OsPcG* genes were unevenly distributed on the nine chromosomes of rice, with more genes on chromosomes 1, 3, 8, and 9, of which four members were found on Chr03, three on Chr01, Chr08, and Chr09, and only one gene on chromosomes 2, 4, 5, 6, and 10. On chromosome 8, *OsPcG5* and *OsPcG6* formed a gene cluster, suggesting that they may have originated from a gene duplication event. *OsPcG7* and *OsPcG12* were separated by 500 kb on chromosome 4, whereas *OsPcG9* and *OsPcG10* were spaced more than 2000 kb apart on chromosome 1, suggesting that these genes may have undergone different evolutionary patterns. It was observed that most of the *OsPcG* genes were localized in the arm region of the chromosome, while *OsPcG4* and *OsPcG14* were mainly concentrated in the proximal mitotic region of the chromosome, which might be affected by different transcriptional regulatory mechanisms. In addition, we also performed chromosomal localization in other species (Figure S1).

### 2.2. Physicochemical Properties and Predicted Subcellular Localization of OsPcG Proteins

The amino acid length of this family of proteins ranged from 376 to 1607 amino acids (aa) (Table 1). The relative molecular weight (MW) ranged from 42.04 to 181.5 kDa, the isoelectric point (pI) ranged from 4.77 to 9.37, the instability index ranged from 44.39 to 69.16, and the aliphatic index ranged from 63.22 to 86.33. The isoelectric point (pI) ranged from 4.77 to 9.37, the Instability Index (II) ranged from 44.39 to 69.16, and the aliphatic index (AI) was distributed between 63.22 and 86.33. Physicochemical analysis showed that all OsPcG proteins exhibited hydrophilic characteristics (GRAVY values less than 0), suggesting that they may exist in the cytoplasm or nucleus in a soluble form. In addition, the instability index of all OsPcG proteins was greater than 40, suggesting that they may be unstable and susceptible to degradation in the cell [40]. In terms of isoelectric point, the pI values of most proteins ranged from 5.5 to 9.2, suggesting that they may be more stable in neutral to weakly alkaline environments. The lipid index analysis, on the other hand, showed that the heat resistance of different OsPcG proteins varied. For example, the lipid indices of *OsPcG5* and *OsPcG7* were both greater than 80, suggesting that they may be more heat-resistant [41], whereas members such as *OsPcG1*, *OsPcG11*, and *OsPcG14* had lower lipid indices, suggesting that they may be more susceptible to conformational changes or denaturation under high-temperature conditions [42]. These differences in physicochemical properties may affect the stability of OsPcG proteins and their functions in cells.

Predictions of subcellular localization indicate that OsPcG proteins function in different cellular structures. Among them, one member (6.7%) was localized in the cytoplasmic membrane, which may act as a transmembrane receptor or be involved in signaling. The majority of OsPcG proteins (11 members, 73.3%) were localized in the nucleus, which is consistent with the role of PcG proteins in epigenetic regulation and suggests that they may be involved in histone modification and gene silencing. Three members (20.0%) were localized in the cytoplasm, suggesting that they may act as regulators and transfer to the nucleus to perform their functions under specific physiological conditions. In addition, two members functioned in multiple cellular compartments, which may be affected by specific signals or regulatory factors and function in different biological processes. In addition to the members that are predominantly located in the nucleus and cytoplasm, some OsPcG proteins are localized in other cellular compartments. For example, one member (6.7%) is located in chloroplasts, which may be involved in the regulation of photosynthesis, and another member (6.7%) is located in the extracellular matrix, which may be involved in intercellular signaling or the regulation of structural stability. It is noteworthy that no *OsPcG* members were found to be located in the mitochondria, suggesting that their main functions may not be directly related to energy metabolism.

### 2.3. Classification of OsPcG and Phylogenetic Analysis of the Phylogenetic Tree

**Figure 2:**
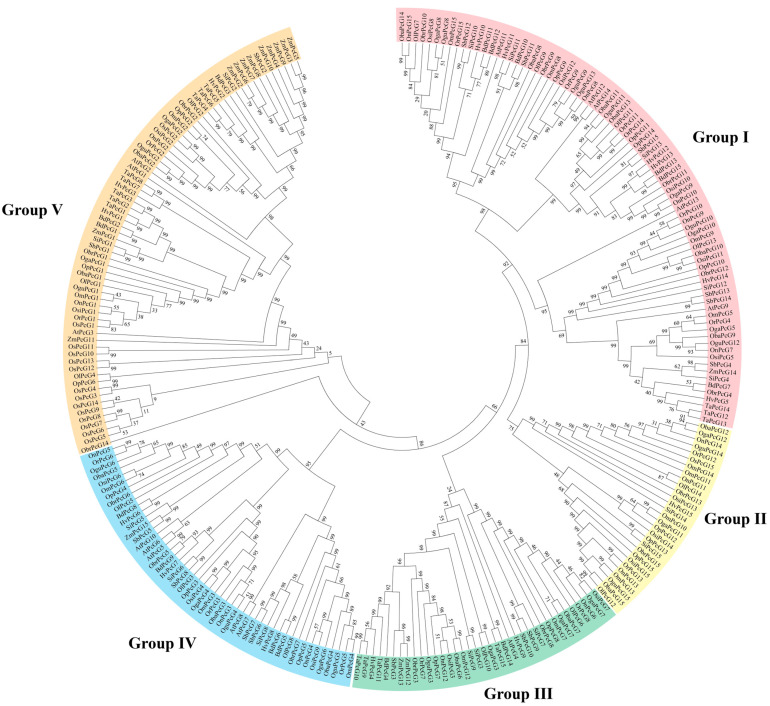
Phylogenetic development of *OsPcG*. Five different colors represent different groups.

Phylogenetic analysis of the PcGG gene family was carried out using the neighbor-joining method (Figure 2). The PcG proteins were classified into five subgroups, including Group I (consisting of 81 out of 265 members, 81/265), Group II (29/265), Group III (37/265) and Group IV (49/265), and Group V (69/265). The classification was primarily supported by the clear topological structure of the tree and high bootstrap values at major nodes. Each subgroup contained PcG proteins from rice as well as other species, indicating their evolutionary conservation. By analyzing the structure and branch morphology of evolutionary trees, we can reveal how species gradually diverged from a common ancestor to form different groups. Each branch node represents a common ancestor, and species begin to diverge and develop their own characteristics after this node. The length of branches in a tree is often used to reflect the evolutionary distance between species, with longer branches indicating greater genetic differences between species and shorter branches indicating closer phylogenetic relationships. Additionally, the overall structure of an evolutionary tree visually illustrates the evolutionary process of species along different evolutionary pathways, with each branch representing a specific species or group of species, further revealing their phylogenetic relationships and evolutionary history.

### 2.4. Structural Analysis of Rice OsPcG

**Figure 3:**
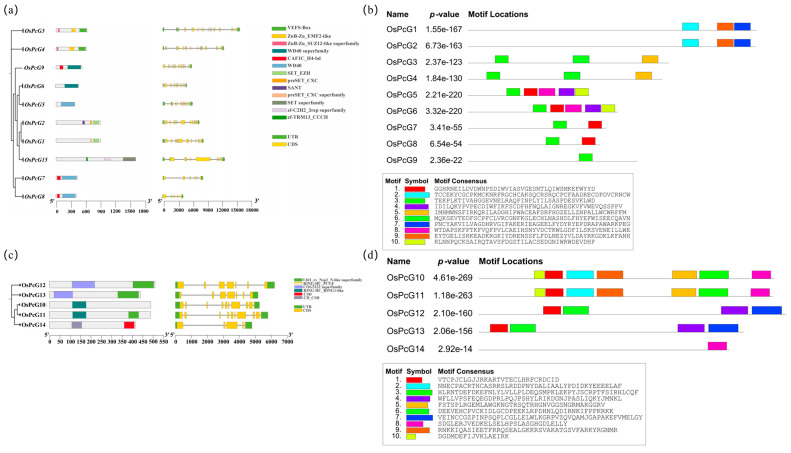
Protein and genomic structural analysis of rice *OsPcGs*: (**a**) PRC2 complex gene structure and functional structural domain analysis; (**b**) PRC1 complex gene structure and functional structural domain analysis; (**c**) PRC1 complex motif assay; (**d**) PRC2 complex motif assay.

In the study of PcG proteins, we identified 10 different motifs, named Motif 1 to Motif 10 (Table S2). The analysis showed that two (13.3%) PcG proteins contained only one motif, two (13.3%) proteins contained two motifs, and 11 (73.3%) proteins contained three or more motifs. In the motif prediction of Polycomb Repressive Complex 1 (PRC1), Motif 1 had the highest frequency, indicating high sequence similarity among PRC1 gene members. In addition, Motifs 2, 5, 6, 9, and 10 appeared only in *OsPcG10* and *OsPcG11*, both of which have RING functional structural domains and show a high degree of intraspecific evolutionary developmental similarity. Similarly, Motifs 3, 4, and 7 were found only in *OsPcG12* and *OsPcG13*, which also showed a high degree of consistency in their functional structural domains and evolutionary developmental features, implying a high degree of homology between the two. Notably, *OsPcG14* was not found to have the same motif structure as the other members, so it was hypothesized that it might be the only homolog of Polycomb (Pc) in rice. In the motif prediction of Polycomb Repressive Complex 2 (PRC2), several genes shared common motifs and exhibited highly similar functional domains. Further analysis showed that these genes also displayed comparable levels of statistical significance (*p* values) in their expression patterns, indicating potential associations among them. Based on these results, we hypothesize that the 15 screened genes are likely to be members of the rice PcG family (Figure 3a,b). In terms of gene structure, the number of exons of *PcG* genes ranged from 5 to 20 and the number of introns ranged from 4 to 21. Among them, eight members (57.1%) had 5 to 10 exons, and nine members (62.3%) contained 4 to 10 introns. The genes with the highest number of exons and introns were *OsPcG3* and *OsPcG4*. *OsPcG4* contained 20 exons and 19 introns, while *OsPcG3* contained 20 exons and 21 introns. Genes with the same number of exons were grouped together, suggesting that there may be some correlation between gene structures and their clustering relationships. Further observation revealed that genes with similar structures also showed a high degree of consistency in the number of exons and introns in their genomes.

### 2.5. cis-Acting Elements in the OsPcG Promoter Region

**Figure 4:**
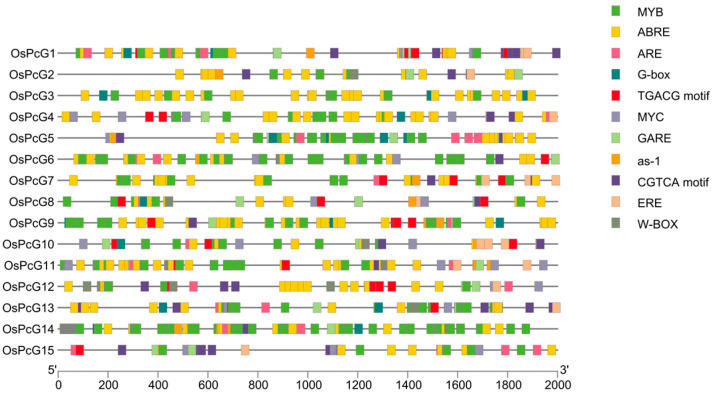
Analysis of *cis*-acting elements of the *OsPcG* promoter sequence.

In order to predict the potential functions of the *OsPcG* gene family, this study analyzed the *cis*-acting elements in the promoter regions of different genes in its 2000 bp sequence upstream of the CDS. The *cis*-acting elements of all members can be mainly categorized into three classes (Table S3 and Figure 4). The first class of *cis*-acting elements is closely related to the response of plant hormones, including TCA elements, TGACG-motif, MBS, and as-1. The second class of elements is related to plant growth and development and involves elements including, but not limited to, ARE, Box-4, the GT1-motif, ABRE, and the TGACG-motif. The third class of *cis*-acting elements, on the other hand, is associated with plant responses to various stresses, such as MYB, MYC, ERE, and ABRE. By analyzing the distribution patterns of *cis*-acting elements, it is hypothesized that members of the *OsPcG* gene family are closely related to plant growth and development and may be responsive to a variety of stress conditions. In particular, among the multiple transcription factor binding sites identified, these sites may be highly correlated with the functions of gene family members. Elements such as MYB and ABRE were statistically found to occur at a high frequency, and MYB transcription factors are known to be involved in regulating processes such as photosynthesis, the stress response, and secondary metabolism in plants. This finding suggests that this gene family may play an important role in regulating metabolism, photomorphogenesis, and the stress response in plants. In addition, abscisic acid response element (ABRE) was enriched in the promoters of several gene family members, indicating that these genes may be regulated through the abscisic acid signaling pathway under environmental conditions such as drought and salt stress. These responsive progenitors were similarly found when *cis*-acting elements were analyzed in other species (Figure S2). Further experimental studies are expected to validate the expression regulation of these genes under adversity stress, thus providing more evidence to understand the role of the *OsPcG* gene family in adversity response.

### 2.6. Analysis of Intra- and Inter-Species Covariance in the Rice PcG Family

Three syntenic gene pairs were identified by intraspecies covariance analysis of the rice (*Oryza sativa*) *PcG* gene family. The results showed that several genes of this gene family exhibited significant covariance on Chromosome 1 and Chromosome 3. Among them, some genes were located in the colinear regions of different chromosomes, suggesting that the expansion of the *PcG* gene family was mainly driven by whole-genome duplication (WGD) or segmental duplication [39]. In addition, some members showed tandem duplication (TD) patterns, suggesting that localized gene expansion may be achieved through the tandem duplication mechanism [43].

**Figure 5:**
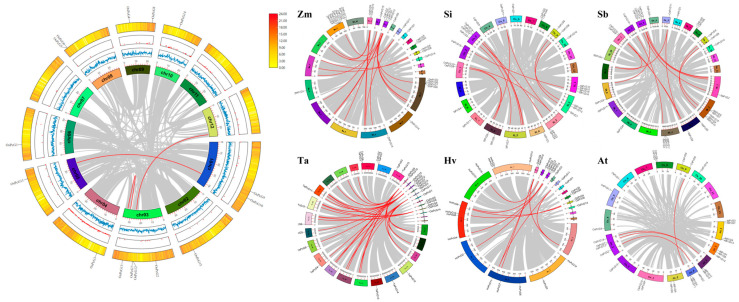
Analysis of *OsPcG* intraspecific and interspecific covariance.

Furthermore, we conducted cross-species covariance analysis of rice with wheat (*Triticum aestivum*), maize (*Zea mays*), sorghum (*Sorghum bicolor*), barley (*Hordeum vulgare*), Arabidopsis (*Arabidopsis thaliana*), and other rice populations (Figure 5 and Figure S3) to explore the cross-species covariance of the PcG gene family with the other rice populations (Figure 5 and Figure S3, Table S4). The analysis showed that the *PcG* gene family is evolutionarily conserved in different species. The analysis showed that the gene family maintained a high degree of covariance among *Poaceae* species, suggesting that its function is relatively stable in *Poaceae* and may perform similar biological functions among different species. However, the covariance between rice and *Arabidopsis thaliana* was more limited compared to that of the grass family, with only a few covariant gene pairs detected. This difference may be attributed to the early phylogenetic divergence between *Gramineae* and *Cruciferae* (*Brassicaceae*) and the subsequent rearrangement of genome structure with gene loss [44]. The diversity of these gene arrangements provides potential key genomic regions for subsequent functional studies with important reference value.

### 2.7. Estimation of Covariance and Ka/Ks Ratio of OsPcG

In order to deeply explore the duplication events of *OsPcG* and its evolutionary relationship, we calculated the Ka/Ks ratio of covariate gene pairs within the species (Table 2). The results show that there are three pairs of duplicated genes with valid Ka, Ks, and Ka/Ks values. The Ka/Ks ratios of all gene pairs were less than 1, indicating that they were mainly subjected to purifying selection and mutations were restricted to maintain the stability of protein function. Among them, the lowest Ka/Ks value (0.23) was found for the co-linear gene pairs located on Chromosomes 1 and 6, suggesting that they have been subjected to more stringent functional constraints during the evolutionary process, and may play a key role in the growth and development of rice.

### 2.8. Analysis of the Expression Pattern of OsPcG Gene in Roots and Leaves

**Figure 6:**
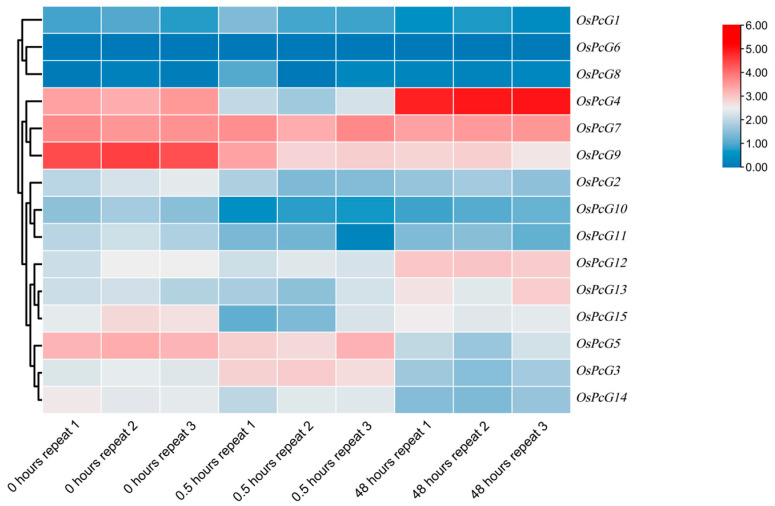
Expression pattern of *OsPcG* gene in roots.

**Figure 7:**
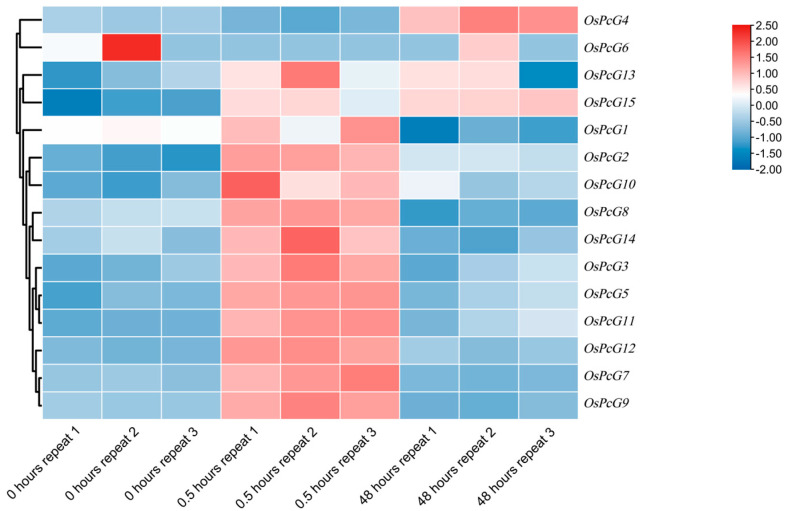
Expression pattern of *OsPcG* gene in leaves.

**Figure 8:**
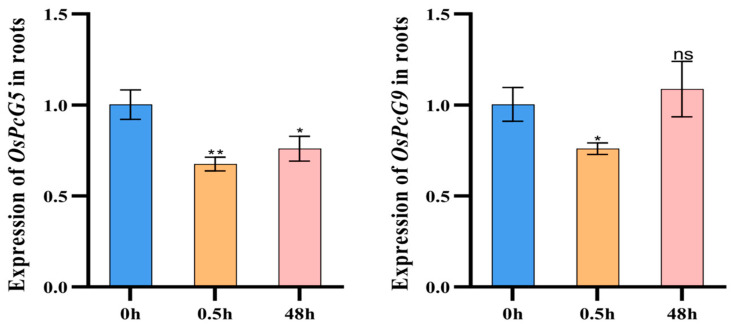
Expression patterns of *OsPcG5* and *OsPcG9* genes in rice roots under salt treatment. The *X*-axis indicates the treatment time points (0 h, 0.5 h, and 48 h), and the *Y*-axis indicates the relative expression levels determined by qRT-PCR. Data are presented as mean ± standard deviation (SD) from three independent biological replicates. Statistical significance was assessed using one-way ANOVA followed by Tukey’s test. “*” and “**” indicate significant differences at *p* < 0.05 and *p* < 0.01, respectively, and “ns” indicates no significant difference.

By analyzing the RNA-seq data of the *OsPcG* gene family, we investigated the gene expression changes in the rice root system after 0 h, 0.5 h, and 48 h of salt-stress treatment (Table S5). Based on the trend of the up- or down-regulation of gene expression, we initially screened out the gene member *OsPcG4*, which was significantly up-regulated in the root system, as well as the members *OsPcG5* and *OsPcG9* (Figure 6), which were down-regulated in expression. *OsPcG5* was studied previously, and named as *OsFIE2*. In contrast, the RNA-seq analysis of the leaves showed that most of the genes were up-regulated in expression after 0.5 h, and all of them showed a trend of down-regulation after 48 h (Figure 7). We also identified the genes that were significantly up-regulated in the root system, such as the *OsPcG5* gene, which was up-regulated in the root system. After further screening, we finally identified *OsPcG5* and *OsPcG9* as candidate genes for in-depth study. *OsPcG9* regulates DNA methylation and siRNA accumulation at the downstream sites of the RdDM pathway by interacting with the RDM15 protein [45,46]. *OsPcG5*, on the other hand, acts as a catalytic multi-comb of H3K27me3 inhibitory complex 2 (PRC2); mutations in its phosphorylation site affect genome-wide H3K27me3 levels, triggering transcriptome changes that ultimately lead to abnormalities in plant organs [47]. To verify the reliability of the RNA-seq data, we further analyzed the expression levels of *OsPcG5* and *OsPcG9* by qRT-PCR (Figure 8) The qRT-PCR results of *OsPcG5* showed an overall decreasing trend in expression, which was consistent with the RNA-seq results. However, the qRT-PCR results of *OsPcG9* did not show a decreasing trend as indicated by the RNA-seq data, but instead showed a certain degree of up-regulation. Therefore, in the follow-up study, we focused only on the functional resolution of *OsPcG5*.

### 2.9. Subcellular Localization of OsFIE2

**Figure 9:**
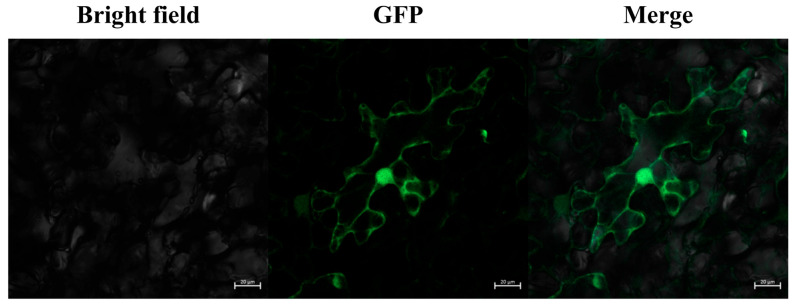
Subcellular localization of *OsFIE2*. Images represent Bright field, GFP, and merged channels. Scale bar = 20 µm.

Observation of GFP fluorescence signals in tobacco leaf cells using laser confocal ultra-high-resolution microscopy (LSM980) showed that the GFP-tagged protein is predominantly localized in the nucleus and cytoplasm (Figure 9). Localization in the cytoplasm suggests that the protein may participate in intracellular signaling or transport processes, potentially mediating cellular responses to developmental cues or environmental stimuli. Its nuclear localization implies a possible role in transcriptional regulation, either as a transcription factor or a regulatory component influencing the expression of downstream target genes. Taken together, the dual localization of this protein in the nucleus and cytoplasm indicates that it may function in both signaling transduction and gene regulatory networks, providing an experimental foundation for further investigation of its molecular mechanism and biological roles.

### 2.10. Role of FIE2 Gene in Regulating Plant Height in Transgenic Rice

To further elucidate the function of OsFIE2, we employed CRISPR/Cas9-mediated gene editing using a target-specific sgRNA (5′-CGTGCAACAAGCTCACCGAGTTTTAGAGCTAGAAATAGCAAGTTAAAATAAGGCTAGTCCGTTATCAACTTGAAAAAGTGGCACCGAGTCGGTGC-3′). However, no viable homozygous mutants were recovered, suggesting that complete loss of *OsFIE2* function is lethal—consistent with previous reports highlighting its indispensable role in rice development [48,49].

**Figure 10:**
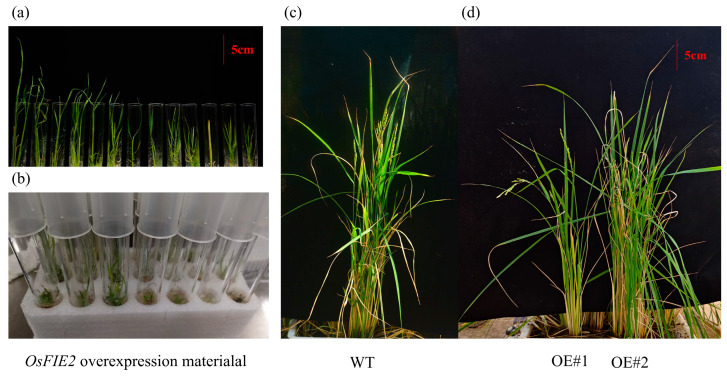
*OsFIE2* overexpression material. (**a**,**b**) Growth performance of OsFIE2 overexpression seedlings at the early seedling stage, showing developmental differences compared with the wild type. (**c**) Wild-type (WT) rice plants at the mature stage, exhibiting normal growth and height. (**d**) OsFIE2 overexpression lines (OE#1 and OE#2) at the mature stage, displaying reduced plant height relative to WT. Scale bar = 5 cm.

Given the inability to obtain knockout lines, a functional analysis was conducted using overexpression materials. An *OsFIE2* construct carrying an N-terminal FLAG tag, driven by a constitutive promoter, was introduced into the *Oryza sativa cv*. Nipponbare background via *Agrobacterium-mediated* transformation. Phenotypic evaluation revealed notable alterations in plant height. While some transgenic seedlings exhibited marked growth inhibition and developmental arrest during the early seedling stage (Figure 10a,b), others continued to grow, but remained significantly shorter than wild-type controls at maturity (Figure 10c,d). A subset of individuals achieved near-normal stature, but still displayed subtle deviations in height, indicating variability in phenotypic expression among overexpression lines. These observations suggest that *OsFIE2* influences vegetative growth, likely by modulating pathways involved in cell elongation or meristem activity. The presence of distinct height differences at early and mature stages underscores its role in regulating developmental dynamics during the vegetative phase. As a core component of the Polycomb Repressive Complex 2 (PRC2), *OsFIE2* contributes to the transcriptional repression of key developmental genes and plays a critical role in maintaining epigenetic homeostasis throughout both vegetative and reproductive growth. Furthermore, previous studies have demonstrated functional divergence between *OsFIE2* and its homologs, with *OsFIE2* being essential for embryo viability and proper seed formation [48,49].

### 2.11. OsFIE2 Overexpression Enhances H3K27me3 Accumulation

**Figure 11:**
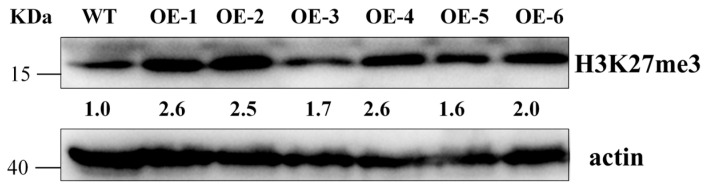
Western blot results of *OsFIE2* overexpression material.

To examine whether *OsFIE2* influences H3K27me3 accumulation in rice, Western blot analysis was conducted using anti-H3K27me3 antibodies in *OsFIE2* overexpression (OE) lines (Figure 11). Four OE lines (OE-1, 2, 4, 6) displayed significantly elevated levels of H3K27me3 compared to the wild type (WT), indicating that *OsFIE2* overexpression promotes H3K27me3 deposition. Actin served as a loading control to ensure equal protein input across samples.

These results confirm the functional role of *OsFIE2*, a key component of the PRC2 complex, in mediating H3K27me3 modification. The increased enrichment of this repressive histone mark is consistent with the established role of PRC2 in transcriptional silencing and suggests that *OsFIE2* contributes to gene repression through epigenetic regulation. This regulatory mechanism likely underpins the developmental alterations and stress-responsive phenotypes observed in transgenic lines, supporting the broader role of PcG proteins in plant growth and environmental adaptation.

## 3. Discussion

This study provides valuable insights into the role of Polycomb group (PcG) proteins, specifically *OsFIE2* (previously referred to as *OsPcG5*), in modulating stress responses and developmental processes in *Oryza sativa* under salt stress conditions. By identifying 15 *OsPcG* genes within the rice genome, our understanding of this gene family has significantly advanced. These genes exhibit evidence of gene duplication and conserved motifs, implying substantial evolutionary divergence and functional diversification [2,3]. The *OsPcG* genes are evolutionarily conserved and appear to play pivotal roles in regulating plant growth and adaptation to abiotic stresses like salinity [4]. Chromosomal analysis uncovered an uneven distribution of *OsPcG* genes across rice chromosomes, with notable clustering on Chromosomes 1, 3, 8, and 9. This suggests that, beyond whole-genome duplications, segmental and tandem duplications have contributed to shaping the evolution of the *OsPcG* family [5]. As observed in other plants, gene clustering often correlates with genomic duplications, which may explain the functional diversification observed within the *OsPcG* gene family. Additionally, the conserved motifs shared between rice and *Arabidopsis* reinforce the notion that PcG proteins play a conserved role in regulating developmental processes and stress responses [6]. A particularly important finding from this study is the identification of *OsFIE2* as a crucial gene in the response to salt stress. Transcriptomic analysis, validated by qRT-PCR, demonstrated that *OsFIE2* expression is notably downregulated under salt stress in both roots and leaves, suggesting its potential role as a key repressor in the plant’s stress response network [7,8]. The dual localization of *OsFIE2* in the nucleus and plasma membrane implies that it may have dual functions: regulating gene expression within the nucleus while also participating in stress signaling pathways. This finding aligns with the existing literature on other PcG proteins, which are involved in stress adaptation in *Arabidopsis* [9]. Furthermore, the increased accumulation of H3K27me3 in *OsFIE2*-overexpressing lines suggests that *OsFIE2* contributes to transcriptional repression, affecting plant height and development under both normal and stress conditions [49]. These findings provide a foundation for future research on the functional characterization of *OsFIE2* and its interaction with other chromatin-remodeling complexes. Investigating the integration of *OsFIE2* regulation with other plant epigenetic networks, such as those controlled by MYB and ABRE transcription factors, could provide new insights into how plants orchestrate growth and stress adaptation [50]. Future studies should also explore the role of *OsFIE2* in response to other abiotic stresses, such as drought and heat, which could reveal additional mechanisms relevant for enhancing crop resilience. Additionally, identifying epigenetic markers like H3K27me3, which are involved in gene repression and stress tolerance, could help in the development of new strategies for breeding salt-resistant rice varieties [13,51]. In summary, the *OsFIE2* gene plays an essential role in regulating growth and development in rice, especially under stress conditions. This research contributes to the expanding field of plant epigenetics, underscoring the potential of *OsFIE2* as a target for improving stress tolerance in crops through genetic and epigenetic approaches [52].

## 4. Materials and Methods

### 4.1. Identification of Members of the PcG Gene Family in Oryza sativa and Chromosome Localization

To identify PcG family members in rice, we performed a Blastp search using the known amino acid sequence of the *Arabidopsis* PcG protein as a query https://blast.ncbi.nlm.nih.gov/Blast.cgi, accessed on 12 April 2024 The *Arabidopsis* sequence was obtained from the TAIR database https://www.arabidopsis.org/, accessed on 12 April 2024 Rice complete genome data were obtained from EnsemblPlant https://plants.ensembl.org/Oryza_sativa/Info/Index, accessed on 12 April 2024. Using the program edited by Chen et al. [53], redundant sequences were removed from the rice genomic data using Fasta Stats and Genome Length Filter in the software TBTOOLS II (version 2.088). We used HMMER v3.4 https://www.ebi.ac.uk/Tools/hmmer/, accessed on 12 April 2024. to detect the conserved structural domains of the PcG protein family with a query threshold of E-value ≤ 1 × 10^−10^. Next, the Batch Network CD search tool https://www.ncbi.nlm.nih.gov/Structure/bwrpsb/bwrpsb.cgi, accessed on 12 April 2024 was used to further examine the conserved structural domains of candidate PcG members. Family members are named primarily on the basis of homologous sequences to *Arabidopsis thaliana*, or on the basis of their chromosomal location if more than one homologous gene is found in *Arabidopsis thaliana*. Positional information of PcG family members was obtained from the rice genome sequence using the program in the software R Studio (version 1.3.959). This includes chromosome position, length, and related annotation information. A basic physical map describing the location and distribution of the PcG gene family was drawn using the Gene Location Visualize program in TBTOOLS II (version 2.088) with default parameters [53].

### 4.2. Predicting the Physicochemical Properties and Subcellular Localization of PcG Proteins

Physicochemical properties of the entire gene family were predicted using the ProtParam ExPASy server https://www.expasy.org/, accessed on 14 April 2024 [54], including properties such as amino acid number, molecular weight, isoelectric point (pI), instability index, lipid index, lipophilicity, and hydrophilicity. The subcellular location of the PcG protein was predicted using Cello http://cello.life.nctu.edu.tw/, accessed on 14 April 2024 [55].

### 4.3. Gene Structure Analysis and Phylogenetic Development

Using the program developed by Chen et al. [54] in combination with the BLAST Zone function of TBtools II (version 2.088), we performed BLAST searches with the known amino acid sequences of OsPcG proteins as queries. With an E-value cutoff of ≤1 × 10^−10^, we identified 250 PcG members across 17 additional species. After removing redundancy and consolidating their amino acid sequences, a total of 265 PcG members were ultimately obtained. Phylogenetic analysis of *OsPcGs* was performed using MEGA X(bootstrap = 1000 replicates) [56]. The conserved motifs of PcG proteins were predicted using MEME https://meme-suite.org/meme/, accessed on 15 April 2024, and the genome sequences of rice PcG family members were extracted using TBTOOLS II (version 2.088) [53] and analyzed by the online platform Gene Structure Display Server (GSDS) 2.0 https://gsds.gao-lab.org/, accessed on 12 April 2024 to analyze the gene structures of PcG members.

### 4.4. Predicting cis-Acting Elements in Promoter Regions

All promoter sequences 2000 bp upstream of *OsPcG* were screened using the software TBTOOLS II (version 2.088), and the screened sequences were analyzed using PlantCARE https://bioinformatics.psb.ugent.be/webtools/plantcare/html/, accessed on 16 April 2024 to predict the *cis*-acting elements within the promoter region to understand the presence and location of different *cis*-regulatory elements. Finally, a comprehensive map of *cis*-acting elements was constructed with the help of TBTOOLS II (version 2.088) [53].

### 4.5. Collinearity and Estimation of Ka/Ks Ratio Analysis

Genes were analyzed for covariance using One Step McScanX-super Fast (E-value 1 × 10^−5^) in TBtools software (version 2.088). Gene Ka/Ks values were analyzed using the synonymous/nonsynonymous mutation ratio calculator in the software [53]. These analytical tools provide a beneficial aid in explaining gene evolution. The synonymous substitution rate (Ks) can be used to estimate the dispersion time (T) of a gene or genome duplication event, where the dispersion time (T) equals Ks divided by twice the neutral substitution rate (λ).

### 4.6. Plant Material

Plant materials were selected from a typical cultivated rice (*Oryza sativa*) variety, Nipponbare, and placed in an intelligently controlled greenhouse to be cultivated under tightly controlled environmental conditions, with the temperature being maintained at a constant 26 °C. During the growth process, the precise regulation of the photoperiod is crucial for the growth and development of rice. During the nutritive growth stage, the photoperiod was set at 16 h of light/8 h of dark (16:8); after entering the reproductive growth stage, the photoperiod was adjusted to 10 h of light/14 h of dark (10:14). In addition, plants have different water requirements at different growth stages, and the water supply needs to be strictly controlled to ensure optimal growth.

When the plants grew to the tillering stage, 140 mM NaCl was applied for salt-stress treatment and samples were taken at 0 h, 0.5 h, and 48 h of treatment, respectively. The collected samples were rapidly frozen in liquid nitrogen at −80 °C for subsequent RNA extraction and analysis.

### 4.7. Transcript Expression Analysis During Root and Leaf Development in Rice

In this study, the rice (*Oryza sativa* L.) cultivar Nipponbare was selected as the study material for transcriptome sequencing analysis. Total RNA was extracted from different experimental treatments or tissue samples, and the quality and integrity of RNA were examined by a NanoDrop2000 spectrophotometer (Thermo Fisher Scientific, Waltham, MA, USA) and agarose gel electrophoresis. Transcriptome libraries were constructed using the NEBNext^®^ Ultra™ RNA Library Prep Kit (NEB, Ipswich, MA, USA) and subsequently sequenced and processed by the company. The expression pattern of the *OsPcG* gene was analyzed based on FPKM (Fragments Per Kilobase of transcript per Million mapped reads) values. Heatmaps were generated using TBtools software after the data results were taken as a logarithmic number with a base of 2 [53]. Differentially expressed genes (DEGs) were screened for |log2FC| ≥ 1 and *p* < 0.05.

### 4.8. RNA Extraction and qRT-PCR Analysis

Total RNA extraction of rice (*Oryza sativa* L.) samples was performed using TRIzol reagent (Invitrogen, Carlsbad, CA, USA), and its standardized procedure was strictly followed. The concentration and purity of the extracted RNA samples were determined by a NanoDrop 2000 spectrophotometer (Thermo Fisher Scientific, Waltham, MA, USA), and the A260/A280 ratio of the RNA was maintained between 1.8 and 2.0 to ensure that the quality of the samples was in accordance with the requirements of subsequent experiments. Reverse transcription was performed using a SuperScript™ III Reverse Transcriptase Kit (Invitrogen, Carlsbad, CA, USA) to transcribe total RNA into cDNA to be used as a template for qRT-PCR. The specificity of gene CDS sequences was analyzed by the Phytozome database (https://phytozome-next.jgi.doe.gov/, accessed on 20 June 2025) to screen for highly specific target sequences. qRT-PCR primer design was carried out in SnapGene software (version 6.0.2), which requires annealing temperatures in the range of 56–60 °C. The primers were designed in SnapGene software (version 6.0.2), requiring an annealing temperature in the range of 56–60 °C and a GC content in the range of 40–60%, and ensuring that there were no complementary bases at the end of the primer sequences to avoid non-specific amplification. qRT-PCR was performed using the Taq Pro Universal SYBR qPCR Master Mix reagent (Vazyme, Nanjing, China), and the rice endogenous gene *Actin* was used as a standardized reference. The rice internal reference gene *Actin* was used as the standardized reference. The relative expression of the genes was calculated based on the 2^−ΔΔCt^ method. Three biological replicates were included in all the experiments to improve the reliability and reproducibility of the experimental data.

### 4.9. Subcellular Localization of the Rice PcG Family Member OsFIE2

The coding sequence (CDS) of the *OsFIE2* gene was amplified using specific primers, using the cDNA obtained from reverse transcription as a template, and the entry vector was constructed. The LR recombination reaction was performed using the Gateway cloning system, and the *OsFIE2* gene was seamlessly inserted into the fluorescent expression vector (1300-GW-GFP). Positive clones were screened by *E. coli* (TOP10) transformation, and Sanger sequencing was used to verify the correctness of the inserted fragments. The correctly recombinant plasmid was transferred into *Agrobacterium tumefaciens* strain GV3101 and positive clones were screened using antibiotics and cultured in LB medium containing 50 mg/mL rifampicin and 50 mg/mL kanamycin until the OD600 value reached 0.8–1.2. Subsequently, the bacterial solution was added to the resuspension solution (10 mM MgCl_2_, 10 mM MES, 200 μM AS) and left at room temperature for 1–3 h to induce gene expression. *Agrobacterium* solution was injected into the cellular gap of tobacco leaves using syringe injection, and the transfected tobacco plants were incubated in a greenhouse at 25 °C protected from light for 12 h, after which, they were restored to normal light conditions for 24–48 h. The transfected plants were then incubated in a greenhouse at 25 °C protected from light for 12 h, after which, they were returned to normal light conditions. Subsequently, samples were prepared, and GFP fluorescence signals were observed using a laser confocal ultra-high resolution microscope (LSM980) to analyze the expression localization of the *OsFIE2* gene and its interaction.

### 4.10. Generation of OsFIE2 Overexpression Lines in Rice

The full-length coding sequence of *OsFIE2* from *Oryza sativa* L. *cv*. Nipponbare was amplified and cloned into the pENTR vector, then recombined into the destination vector 512-flag-gw via Gateway™ (Invitrogen, Carlsbad, CA, USA) cloning to generate the final expression construct 512-flag-*OsFIE2*. The construct was driven by a constitutive promoter and carried a FLAG epitope tag at the N-terminus. The binary vector 512-flag-*OsFIE2* was introduced into *Agrobacterium tumefaciens* strain EHA105 and subsequently transformed into rice cultivar Nipponbare by *Agrobacterium-mediated* transformation. Transformation and tissue culture procedures were carried out by Boyuan Biotechnology Co., Ltd. (Hangzhou, China).

### 4.11. Western Blot Analysis of H3K27me3 in OsFIE2 Overexpression Lines

To assess whether *OsFIE2* overexpression affects the accumulation of H3K27me3, leaves were harvested from transgenic rice lines overexpressing *OsFIE2* and wild-type Nipponbare lines during the seedling stage. Each sample (0.1 g) was processed using a homogenizer, followed by protein extraction with a protein extraction solution. Equal amounts of total histones (20–30 µg per lane) were separated by 10% SDS-PAGE electrophoresis and transferred to a PVDF membrane (Millipore, Burlington, MA, USA). The membrane was blocked in TBST containing 5% non-fat milk for 1 h, followed by overnight incubation at 4 °C with primary antibodies: anti-H3K27me3 antibody (1:2000, ABclonal, Wuhan, China, catalog number A2363) and plant-specific antibody (1:5000, ABclonal, Wuhan, China, catalog number AC009) as an internal control.

The membrane was then incubated at room temperature with HRP-labeled secondary antibody (1:5000, Woburn, MA, USA) for 1 h. Detection was performed using SuperSignal™ West Atto chemiluminescent substrate (Thermo Fisher, USA), and band signals were imaged using the Tanon 5200 chemiluminescent imaging system (Tanon, Shanghai China). Band intensities were quantified using ImageJ software (version 1.8.0). Compared to the wild-type control group, multiple *OsFIE2*-overexpressing lines showed significantly elevated H3K27me3 levels, indicating that *OsFIE2* plays a role in histone methylation processes associated with transcriptional repression.

## 5. Conclusions

This study offers a detailed examination of the Polycomb group (PcG) protein family in *Oryza sativa*, with a particular focus on *OsFIE2*. We identified 15 *OsPcG* genes, revealing extensive gene duplication and functional diversification. Phylogenetic analysis and motif exploration highlighted conserved elements, while chromosomal mapping indicated evolutionary patterns shaped by segmental and tandem duplications. Functional analysis of *OsFIE2* showed its role in regulating responses to salt stress, with both transcriptomic and qRT-PCR data indicating its downregulation under stress. The protein’s localization in the nucleus and plasma membrane suggests it plays a dual role in gene regulation and stress signaling. Additionally, *OsFIE2* overexpression increased H3K27me3 levels, supporting its involvement in transcriptional repression. These results highlight *OsFIE2*’s crucial role in plant growth and stress tolerance. As an epigenetic regulator, *OsFIE2* may serve as a valuable target for improving crop resilience. Future studies should explore its interactions with other chromatin regulators and its broader involvement in development and stress responses.

## Figures and Tables

**Table 1:** Analysis of physicochemical properties of OsPcG proteins.

Sequence ID	Number of Amino Acid	Molecular Weight	Theoretical pI	Instability Index	Aliphatic Index	Grand Average of Hydropathicity	Prediction of Subcellular Localization
*OsPcG1*	896	100,295.81	8.02	51.46	63.98	−0.747	Nuclear
*OsPcG2*	895	99,862.93	8	51.02	65.74	−0.704	Nuclear
*OsPcG3*	624	71,546.95	5.87	49.48	78.48	−0.502	Nuclear
*OsPcG4*	604	68,615.51	6.52	51.82	76.69	−0.434	Nuclear
*OsPcG5*	376	42,035.94	5.68	44.98	86.33	−0.107	Plasma Membrane/Extracellular
*OsPcG6*	466	51,848.95	7.55	48.48	79.27	−0.295	Chloroplast
*OsPcG7*	428	48,359.93	4.77	47.79	80.21	−0.49	Cytoplasmic/Nuclear
*OsPcG8*	410	44,741.85	4.98	44.39	79.76	−0.363	Cytoplasmic
*OsPcG9*	525	57,532.24	6.24	45.7	70.61	−0.546	Cytoplasmic
*OsPcG10*	490	53,639.04	8.22	59.71	65.18	−0.754	Nuclear
*OsPcG11*	488	53,639.50	5.5	67.1	63.22	−0.867	Nuclear
*OsPcG12*	510	56,251.41	8.86	69.16	65.43	−0.856	Nuclear
*OsPcG13*	439	48,637.46	9.37	54.32	75.49	−0.726	Nuclear
*OsPcG14*	415	45,675.33	4.89	64.84	63.23	−0.954	Nuclear
*OsPcG15*	1607	181,498.29	6.16	47.39	70.83	−0.571	Nuclear

**Table 2:** Results of *OsPcG* ka/ks analysis.

Seq_1	Seq_2	EffectiveLen	AverageS-Sites	AverageN-Sites	Ka	Ks	Ka/Ks	cN	cS	pN	pS
*OsPcG10*	XP_015640607.1	1410	331.58	1078.42	0.22	0.99	0.23	208.5	182.5	0.19	0.55
*OsPcG12*	XP_015620739.1	1377	321.92	1055.08	0.22	0.63	0.34	200.2	137.8	0.19	0.43
*OsPcG13*	XP_015628300.1	1065	251.75	813.25	0.54	1.90	0.28	311.2	173.8	0.38	0.69

## Data Availability

Transcriptome data are available in the SRA with accession number PRJNA639386.

## Supplementary Materials

The following supporting information can be downloaded at: https://www.mdpi.com/article/10.3390/plants14172805/s1, Figure S1: Chromosome mapping analysis of other species; Figure S2: Analysis of cis-acting elements in promoter sequences of other species; Figure S3: Analysis of *OsPcG* interspecific covariance; Table S1: Statistics of physical location of PcG on Oryza sativa L. chromosomes; Table S2: Motif sequence of PcGs; Table S3: Cis-element analysis of *OsPcGs* gene promoters; Table S4: Results of collinearity analysis between Oryza sativa L. and other species; Table S5: Transcriptome expression data of *OsPcGs* in roots and leaves after 0 h, 0.5 h, and 48 h of salt treatment.
