# Supplementary material for: Genome-Wide Analysis of the Rice PcG Gene Family and Its Involvement in Salt Response and Development
Source: Plants (Basel). 2025 Sep 8;14(17):2805. doi: 10.3390/plants14172805 (PMC12431386; doi:10.3390/plants14172805)
Supplement: Supplementary file 1 [file plants-14-02805-s001.zip › supplementary figures.pdf]

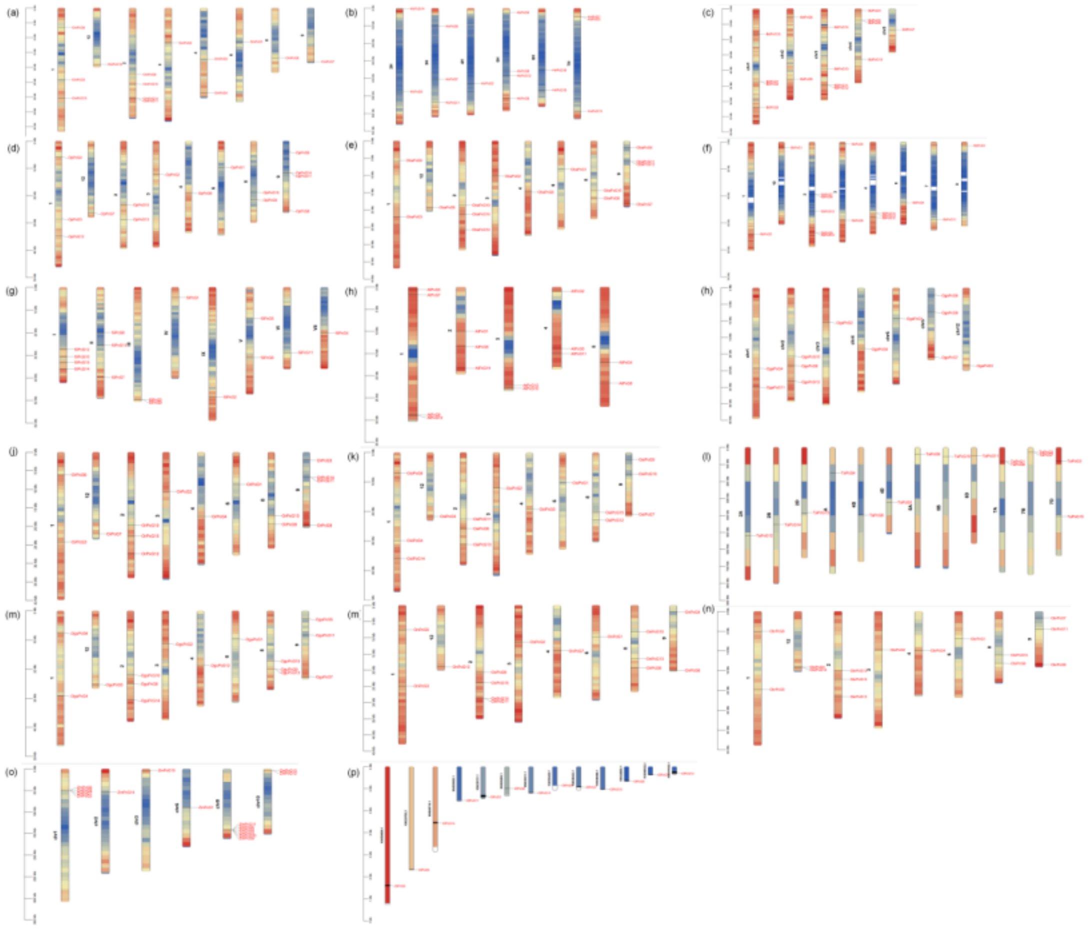

Figure s1 Chromosome mapping analysis of other species

a.Chromosome mapping of Oryza meridionalis b.Chromosome mapping analysis of Hordeum vulgare L c.Chromosome mapping analysis of Brachypodium distachyum

d.Chromosome mapping analysis of Oryza punctata e.Chromosome mapping analysis of Oryza barthii f.Chromosome mapping analysis of Oryza glaberrima

g.Chromosome mapping analysis of Sorghum bicolor h.Chromosome mapping analysis of Setaria italica i.Chromosome mapping analysis of Arabidopsis thaliana

j. Chromosome mapping of Oryza rufipogon k.Chromosome mapping analysis of Oryza sativa Indica l.Chromosome mapping analysis of Triticum aestivum

m.Chromosome mapping analysis of Oryza glumipatula n.Chromosome mapping analysis of Oryza nivara o.Chromosome mapping analysis of Oryza brachyantha

p. Chromosome mapping analysis of Zea mays q.Chromosome mapping analysis of Oryza longistaminata

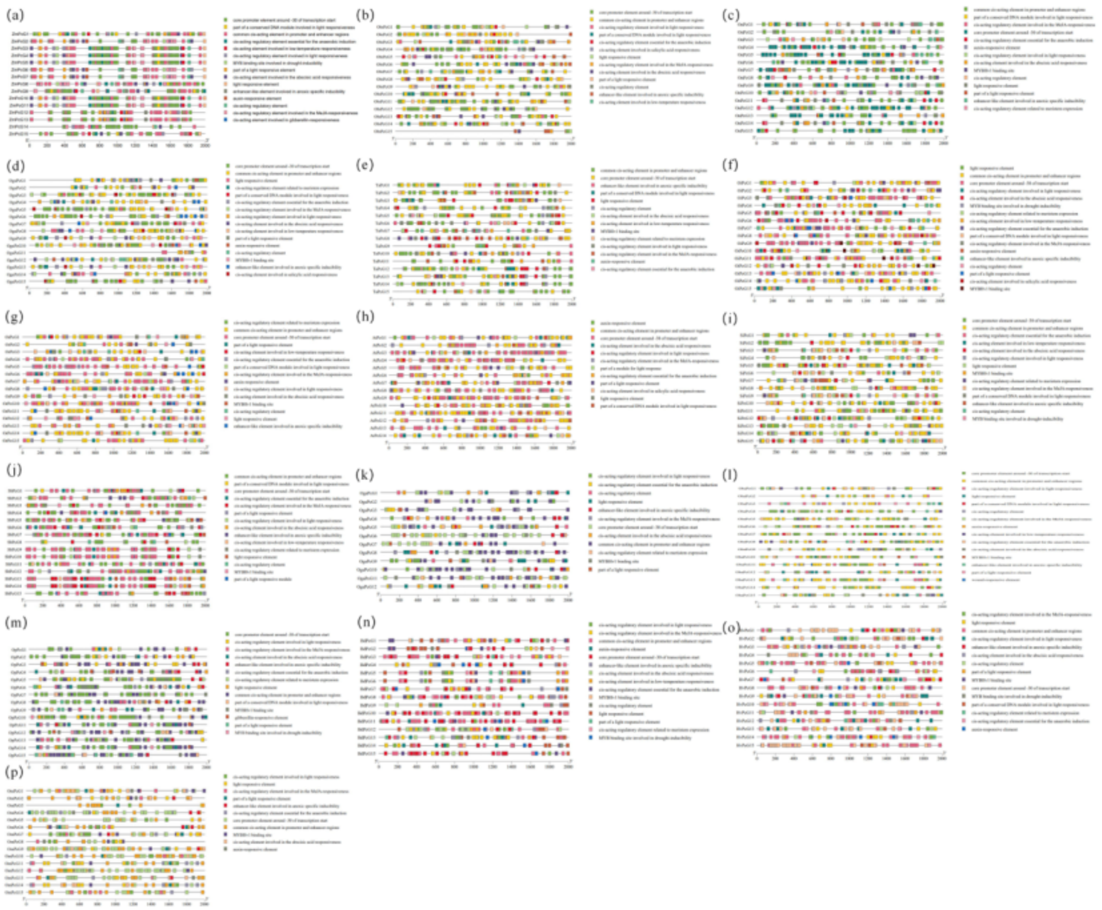

Figure s2 Analysis of cis-acting elements in promoter sequences of other species

a.Analysis of cis-acting elements of the Zea mays promoter sequence

b.Analysis of cis-acting elements of the Oryza brachyantha promoter sequence

c.Analysis of cis-acting elements of the Oryza nivara promoter sequence

d.Analysis of cis-acting elements of the Oryza glumipatula promoter sequence

e.Analysis of cis-acting elements of the Triticum aestivum promoter sequence

f.Analysis of cis-acting elements of the Oryza sativa Indica promoter sequence

g.Analysis of cis-acting elements of the Oryza rufipogon promoter sequence

h.Analysis of cis-acting elements of the Arabidopsis thaliana promoter sequence

i. Analysis of cis-acting elements of the Setaria italica promoter sequence

j.Analysis of cis-acting elements of the Sorghum bicolor promoter sequence

k.Analysis of cis-acting elements of the Oryza glaberrima promoter sequence

l.Analysis of cis-acting elements of the Oryza barthii Indica promoter sequence

m.Analysis of cis-acting elements of the Oryza punctata promoter sequence

n.Analysis of cis-acting elements of the Brachypodium distachyum promoter sequence

o.Analysis of cis-acting elements of the Hordeum vulgare L promoter sequence

p.Analysis of cis-acting elements of the Oryza meridionalis promoter sequence

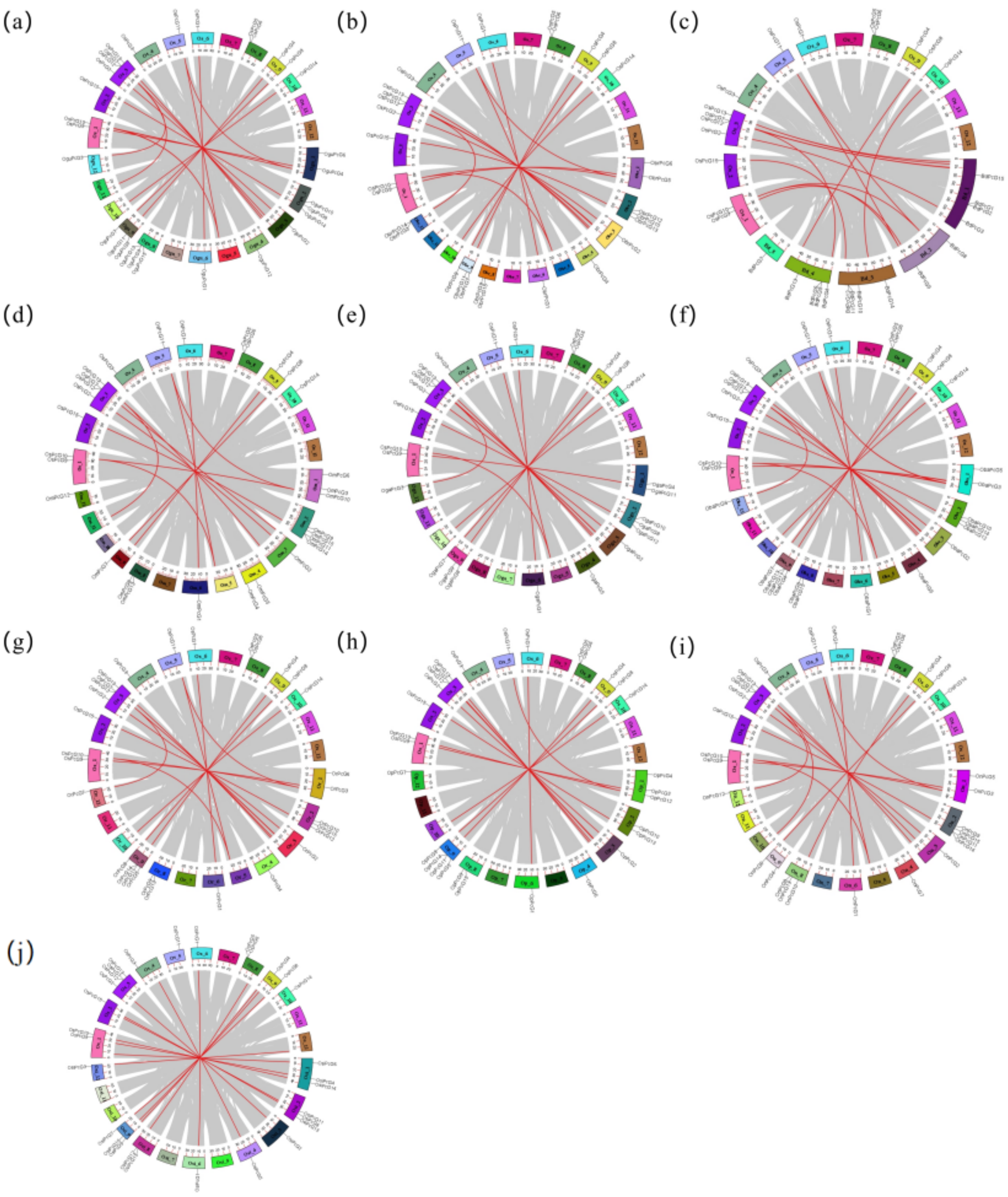

Fig.S3 Analysis of OsPcG interspecific covariance

(a)Collinearity analysis between *Oryza meridionalis* and *Oryza sativaL*

(b)Collinearity analysis between *Oryza brachyantha* and *Oryza sativaL*

(c)Collinearity analysis between *Brachypodium distachyum* and *Oryza sativaL*

(d)Collinearity analysis between *Oryza punctata* and *Oryza sativaL*

(e)Collinearity analysis between *Oryza glaberrima* and *Oryza sativaL*

(f)Collinearity analysis between *Oryza barthii* and *Oryza sativaL*

(g)Collinearity analysis between *Oryza rufipogon* and *Oryza sativaL*

(h)Collinearity analysis between *Oryza glumipatula* and *Oryza sativaL*

(i)Collinearity analysis between *Oryza nivara* and *Oryza sativaL*

(j)Collinearity analysis between *Oryza sativa Indica* and *Oryza sativaL*
